# Welcome to the journal of *Extracellular Vesicles and Circulating Nucleic Acids*: a new open-access scientific journal

**DOI:** 10.20517/evcna.2020.03

**Published:** 2020-12-22

**Authors:** Y. Peng Loh

**Affiliations:** American Biochemist and Molecular Biologist, Bethesda, MD, USA.

A wealth of research has emerged over the last five years showing the importance of extracellular vesicles (EVs) as mediators of intercellular communication. EVs range in size from 30 to 400 nm and according to the size have been classified as microvesicles, exosomes, and oncosomes. EVs are released from cells in normal and pathological conditions, into many body fluids such as blood, urine, saliva, cerebral spinal fluid, and milk. EV contents, which include RNA, proteins, and lipids, reflect the state of the cell of origin, such as during metabolic changes and disease. Therefore, EVs have emerged as potential biomarkers. Furthermore, stem cell EVs have now been found to be important in different types of tissue repair. EVs have also been useful for delivery of siRNA, proteins, and other molecules for therapeutic use, and clinical applications of EVs are emerging. At the same time, studies to better understand the cellular mechanism of EV biosynthesis, trafficking, uptake, and release of EV cargoes in cells have facilitated the production, loading, and purification of EVs for therapy.

Besides EVs, cells release nucleic acids into the circulation and other body fluids, and they are potential biomarkers for disease. Cell-free (cf) DNAs have been used for example to monitor tumor progression and heart transplant rejection, while cf-RNA, especially miRNAs, which are highly stable, are useful biomarkers in cancer and neurodegenerative diseases. To facilitate multi-omics analysis of cell-free nucleic acid biomarkers, new techniques such as electrokinetic chip devices and microfluidic systems have been developed to isolate cf-DNA and exosomes from body fluids, respectively. Liquid biopsy employing circulating exosomes and cf-nucleic acids is a non-invasive and safe alternative to tissue biopsy to monitor disease progression and directing therapy.

The Journal *Extracellular Vesicles and Circulating Nucleic Acids* (EVCNA) provides an online platform for sharing of research data, new methodologies, reviews, and commentaries on these exciting areas of research that are moving at a rapid pace, involving partnerships between academia and industry to bring liquid biopsy biomarker assays and exosomal delivery of therapeutic agents to clinical use. At the same time, the journal supports publication of strong basic research which enables development of clinical applications. EVCNA will be published quarterly with additional Special Issues focusing on specific topics. We have assembled an eminent group of international researchers with expertise across the topics covered by the Journal to serve on the Editorial Board. Manuscripts with clinical relevance are especially encouraged to promote the translation from basic science to clinical applications. The criteria for acceptance will be scientific excellence and originality. We also aim to have a quick turnaround time for processing of articles submitted for publication without compromising rigorous peer review.

In this first issue of EVCNA, scheduled to be launched in December 2020, we will publish a report of the highlights of the American Society of Extracellular Microvesicles 2020 meeting that was successfully held as a virtual conference during 17-19 November 2020 due to the COVID-19 pandemic. We will also include abstracts of talks and posters that were presented at the meeting. Our second issue is scheduled to be published in March 2021 with five scientific articles. We would like to invite you to submit papers for future issues of EVCNA (www.evcna.com).

## Declarations

### Copyright

©The Author 2020.

